# Inner-View of Nanomaterial Incited Protein Conformational Changes: Insights into Designable Interaction

**DOI:** 10.1155/2018/9712832

**Published:** 2018-09-05

**Authors:** Arka Mukhopadhyay, Sankar Basu, Santiswarup Singha, Hirak K. Patra

**Affiliations:** ^1^Karlsruhe Institute of Technology, Institute of Process Engineering in Life Sciences, Germany; ^2^Clemson University, Computational Biophysics Group, Department of Physics and Astronomy, South Carolina, USA; ^3^Department of Microbiology Immunology and Infectious Diseases, University of Calgary, 3280 Hospital Drive NW, Calgary, Alberta, Canada; ^4^Linkoping University, Department of Clinical and Experimental Medicine, Linkoping, Sweden; ^5^Wolfson College, University of Cambridge, Cambridge, UK; ^6^University of Cambridge, Department of Chemical Engineering and Biotechnology, Cambridge, UK

## Abstract

Nanoparticle bioreactivity critically depends upon interaction between proteins and nanomaterials (NM). The formation of the “protein corona” (PC) is the effect of such nanoprotein interactions. PC has a wide usage in pharmaceuticals, drug delivery, medicine, and industrial biotechnology. Therefore, a detailed in-vitro, in-vivo, and in-silico understanding of nanoprotein interaction is fundamental and has a genuine contemporary appeal. NM surfaces can modify the protein conformation during interaction, or NMs themselves can lead to self-aggregations. Both phenomena can change the whole downstream bioreactivity of the concerned nanosystem. The main aim of this review is to understand the mechanistic view of NM-protein interaction and recapitulate the underlying physical chemistry behind the formation of such complicated macromolecular assemblies, to provide a critical overview of the different models describing NM induced structural and functional modification of proteins. The review also attempts to point out the current limitation in understanding the field and highlights the future scopes, involving a plausible proposition of how artificial intelligence could be aided to explore such systems for the prediction and directed design of the desired NM-protein interactions.

## 1. Introduction

**Figure 1:**
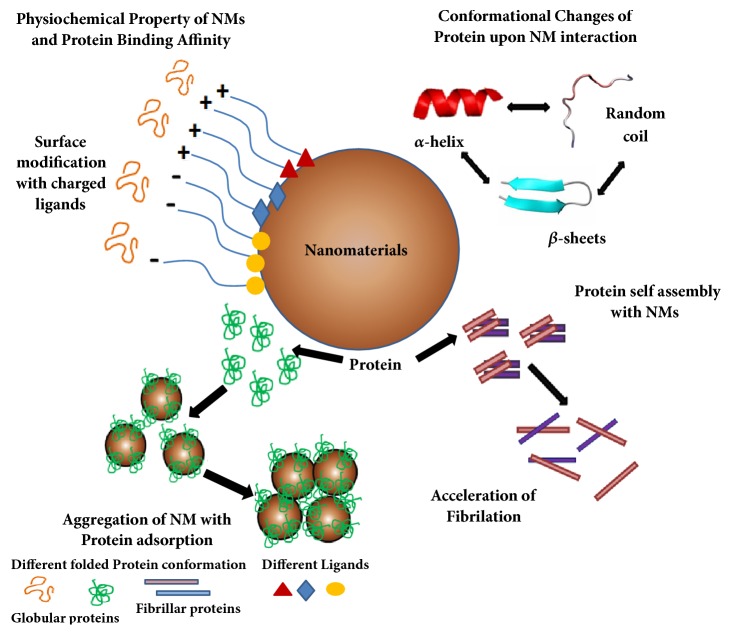
**Overview of NM-protein interaction: cause and effect of nanobiointerface. **Protein adsorption on nanomaterials (NMs) depends upon physiochemical properties of NMs, such as size, shape, and charges on the NMs surface. This is sometimes governed by the charges of the ligands attached on the surface to interact with proteins. Proteins often face conformational changes upon NM interaction. The secondary structure (*α*-helices, *β*-sheets, and random coils) gets perturbed due to interaction with NMs. There is a distinct influence of NMs on self-assembly of proteins. In the presence of NMs, an appropriate conformational change of protein happens which leads to dramatic increase in rate of fibrillation. The binding of proteins onto NMs can lead to NM aggregation associated with protein aggregation.

Over the past few decades, the interaction of nanomaterials (NMs) with biomolecules has become one of the most exciting areas of applied and basic research in bio- and material science. The rapid use of nanobiomaterials in different areas of industries, technologies, and medicine is raising obvious questions about the safe implementation of its prescribed applications [1]. The conjugation of biomolecules with NMs not only introduces biocompatible functionalities into these nanoclusters but also leads to stabilization of the complex (Figure 1). Again, proteins are functionally relevant biomolecules and attachment of NM to protein has found vital applications in catalysis, imaging, and understanding the structural modification of proteins and control of protein catalytic activity with external fields, e.g., magnetic [2]. NM-protein conjugates are not limited to biological applications but rather have outreached fields like material sciences and physical sciences with device design [2] such as biofuel cells and nanosensors.

Compared to bulk materials, the surfaces of NMs have higher free energy owing to their unique electronic distributions, which enable NM surfaces to adsorb proteins preferentially [3]. Evaluation of this change in free energy that occurs during protein adsorption provides the fundamental basis for analyzing the nature of the driving force for the reaction. Free energy change (∆G) can either be entropic or enthalpic or both. While entropic change (∆S) associated with protein adsorption is due to either the dehydration of uncharged surfaces or the removal of the electric double layer from charged surfaces, enthalpic changes (∆H) are mostly due to hydrogen bonding or the formation of donor–acceptor coordination bonds between the protein and the adsorbent surface [3]. The layers consisting of bound or adsorbed proteins around NPs are allegorically called “protein corona” (PC) [4, 5] for their structural resemblance to the original cosmological term, corona, meaning the rarefied gaseous envelope of the sun and other stars. This PC-bilayer then grows rapidly to introduce the bound NM into biological fluid and participate in the exchange of proteins based on their abundance and affinity towards particular NM [5]. One of the main purposes of forming this “layered delivery system” is to prevent any undue cell damage by some NMs (carbon nanotubes, graphene, etc.) [6]. The formation of PC is closely associated with inducing protein conformational changes which have both useful and deleterious effects. Given all these, one of the primary objectives of this review is to recapitulate the physical chemistry of the process as well as the biological impact of PC formation associated with the protein conformational changes, to discuss the current* state of the art, *and to point out the limitations and highlight the future scope.

## 2. Experimental Approaches to Probe Protein-NM Interactions

**Figure 2:**
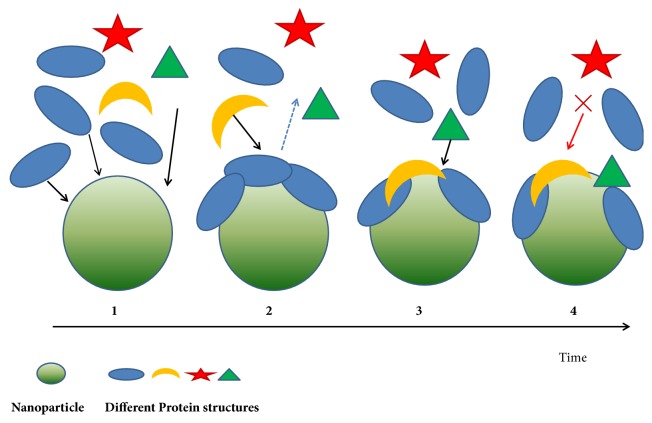
**Nanoprotein corona formation. (1)** An initial corona forms from those proteins (blue) that arrive first to the surface (most abundant protein).** (2)** Initially adsorbed protein with low affinity (blue) is subsequently displaced by a different protein molecule with higher affinity (yellow) arriving later.** (3)** Another molecule (green) which had low affinity initially for the bare surface now adsorbs on the nanoparticle surface owing to favourable with the already adsorbed protein molecules (blue and yellow).** (4)** Another different protein molecule (red) cannot adsorb at all.

There are several experimental techniques to capture changes in size and shape of NMs upon protein adsorption (Figure 2). They include Transmission and Scanning Electron Microscopy (TEM, SEM), Atomic Force Microscopy (AFM), Nanoparticle Tracking Analysis (NTA), Dynamic Light Scattering (DLS), Fluorescence Correlation Spectroscopy (FCS), and Differential Centrifugal Sedimentation (DCS). DLS and FCS determine the thickness of the protein shell by comparison of the hydrodynamic diameter of NPs, which can be measured from their diffusion coefficients (Supplementary) by the Stokes-Einstein equation, before and after the adsorption. Again, since the adsorption of proteins on NPs usually leads to quenching, the changes in NP-fluorescence are also indicative of the formation of a PC [9]. Sometimes, DLS is particularly difficult to apply when NPs are in the size range of proteins (say, <10 nm), as unbound proteins may meddle with. In such troubleshoots, the community has come up with Dielectrophoretic DLS (DDLS) [10] as the alternative where the unwanted signals from unbound proteins are completely suppressed due to the incubation of the PC with complex matrices [9], while on the other end the dissociation coefficients of the NP-protein interactions can be determined using FCS.

The changes in particle size are accompanied by a concomitant change in electrokinetic potential (*ζ*) during the corona formation (Supplementary). As a matter of fact, *ζ*-potential is the only accessible resource to characterize the double layer properties of colloidal systems [11]. The colloidal dispersion exhibits acceptable physical stability when the *ζ*-potential of the particles falls outside the range of −15 to +15 mV. The increased magnitude in *ζ*-potential, in turn, increases the repulsive forces between particles preventing their aggregation. Again, the protein concentration has a direct effect on the hydrodynamic radius of the interacting NPs independent of the *ζ*-potential [12], as revealed by Monopoli et al., in SiO_2_ NPs [2, 9].

One of the major conclusions from virtually all experimental findings was that the PC formation is associated with structural (or conformational) changes in the adsorbed proteins. However, all such studies suffer in their (atomistic) details due to the lack of enough high-resolution experimental structural techniques to be implemented to probe the induced conformational changes. FT-IR is a very sensitive technique to capture the conformational changes as reflected in the vibrational bands corresponding to the amide (or peptide) bonds, also used to probe the covalent adduct formation of Cysteines in the PCs. Again, CD can indirectly capture the structural changes, as revealed in the case of the human iron-protein transferrin upon interacting with SPIONs [13]. Another sensitive indicator is the change in intrinsic fluorescence of a buried tryptophan upon getting exposed to the polar solvent. This can be probed by bis-ANS binding, a common marker for protein unfolding [14]. An example of an effective use of Trp-fluorescence has been demonstrated in probing the CuNP-BSA interaction [15].

## 3. Origin of the Forces Stabilizing Protein Corona and the Induced Protein Conformational Changes

Charged molecular surfaces lead to the formation of an electric double layer when placed in aqueous solutions. The 1st layer of ions gets directly adsorbed on the surface through electrostatic forces leading to the formation of salt-bridges [15], hydrogen bonds [16], charge-dipole, and dipole-dipole interactions while the 2nd layer remains composed of free ions adsorbed to the surface by weaker electrostatic forces (Table 1). Principal forces stabilizing protein corona originate from two fundamental and distinct sources: (i) repulsive forces between interacting proteins help keep nanoparticles in solution (e.g., BSA is used to stabilize gold nanoparticles). However, attractive or depletion forces may dominate at times between proteins so as to drive aggregation of the particles, which further influence change of the size and density of the nanoprotein complexes. (ii) Owing to their unique electronic distributions, the high free energy at the surfaces of nanoparticles (compared to bulk materials) results in absorption of various molecules, most notably proteins. The surface charge on the proteins also triggers electrostatic interactions (hydrogen bonds, salt-bridges, charge-dipole, and dipole-dipole interactions) influencing protein absorption on nanosurfaces. This layer is called the diffused layer as it generally moves in the fluid. The formation of this charged double layer on the NM surface induces local VDW interactions [17] [best explained by quantum electrodynamics [18]], yet another component is known to affect the adsorbed protein's conformations. Sometimes induced charges affect the adsorption of ions onto gold surfaces in the gas phase at a strength similar to chemical bonds while ions and charged peptides in solution are influenced at a strength similar to intermolecular bonds [19]. Hydrogen bonding may further be mediated between polar amino acid residues (e.g., serine, threonine, asparagine, and glutamine) of the adsorbed protein and exposed polar groups (e.g., hydroxyl groups on oxidized metals) on the NM surface [20]. Another major component influencing PC formation and the related conformational changes is the hydrophobic interaction [21] which originates due to the strong antipathy of the nonpolar groups when exposed to a polar environment. Hydrophobic interactions bring about the largest structural changes to the adsorbed proteins as the inner hydrophobic regions of the protein get unfavourably exposed to the solvent. Such an unfavourable exchange was encountered in *α*-lactalbumin, a largely hydrophobic protein, upon interacting with ZnO-NP leading to the deformation of its secondary structures [22]. A similar event has also been reported in case of CNT which when incorporated into the hydrophobic core leads to the destruction of the protein functionality by blocking the active site [23, 24].

## 4. Memory of Protein Corona Formation

PC formation is thought to involve an intrinsic “memory function” [12] which appears to depend on the particular NP interacting with the particular protein sequence. One view addressing the* modus operandii* of this “memory function” hypothesizes that the first protein to get adsorbed on a given NP surface holds the key to develop the “memory” as it is the first protein which has the largest abundance to PC [12, 25]. The “memory” is often developed by means of a conformational change induced on this first adsorbed protein, which, in turn, influences the subsequent (specific) protein-protein interactions constrained by the modified growing surface of the NP-PC assembly [12, 25]. Similarly mechanisms invoke an NP-induced conformational change on the first adsorbed protein; an NP-PC assembly can also potentially get access to otherwise inaccessible endogenous and exogenous substances by biomimicry [12, 26]. This feature has in fact been used as a strategy for selective targeting of drugs, say, by cloaking NPs on the membranes of certain immune cells, thereby providing a shielding effect against an undesired antigen rejection [12, 27].

## 5. A Surface View of the Protein-NM Interaction

**Figure 3:**
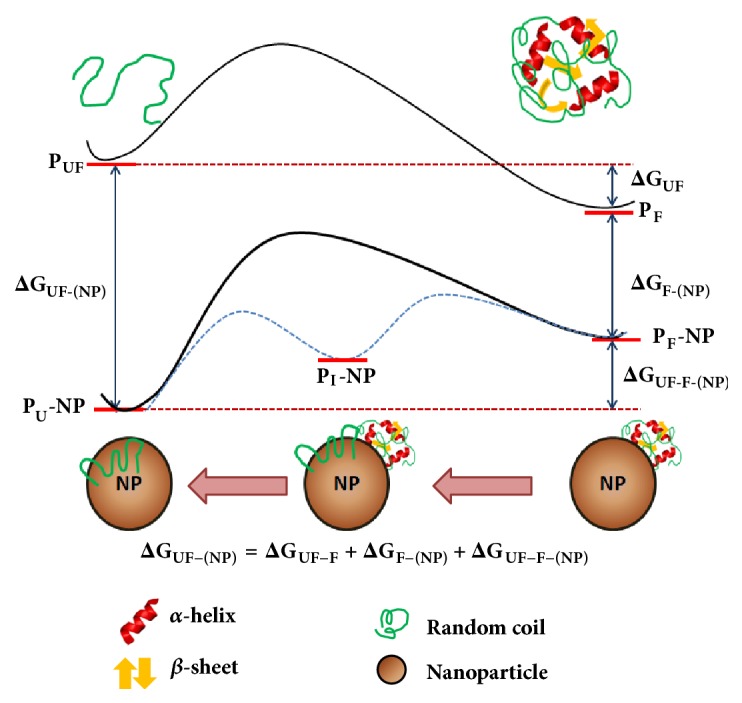
**Free energy profiles of the protein–NP interaction**. A schematic representation of the energy profiles of the protein–NP interaction and its influence on the folding of the protein. From the thermodynamics point of view, the native or folded state of protein (PF) is only marginally more stable than the unfolded state (PUF) physiologically. The binding energy of PUF with a NP is usually larger than that of PF. Correspondingly, the PUF–(NP) complex is usually more stable than the PF–NP complex. From the equation in the diagram it can be shown that larger free energy change of the binding between the folded protein and the NP ( ΔG F-NP ) means a smaller free energy change of the unfolding of the bound protein on the NP surface ( ΔG UF-F-NP ).

One of the key attributes of NMs per se is their “unique” surface properties, arising due to the relative distribution of core and surface electrons, leading to a high surface area-to-volume ratio in comparison with bulk material [28]. In fact, this serves as one of the most fundamental points of discrimination between NMs and bulk matters and forms the basis of the widespread biomedical applications of NMs [29]. Again, one way to look at the protein-NM interface is to treat the interaction between two surfaces of distinctly different origin and scale. To that end, the protein surface has been traditionally well defined and categorized as solvent accessible surfaces [30], VDW surfaces [31, 32], and molecular Conolly surfaces [33] with commonality as well as uniqueness among the different surfaces. So to say, the solvent accessible surface in proteins has found a wide array of applications over the years in the structure dependent functional characterization while the VDW and molecular Connolly surfaces have been particularly useful to study internal packing within folded globules, extending the exercise to facilitate protein design [34]. Again, by definition the molecular surface being smoother than a corresponding VDW surface appropriately tunes the numerical ranges of the related shape descriptors (Sc: shape correlation) as applicable to the packing studies [31, 32, 34]. On the other hand, the NM surfaces of diverse origins can be adequately described by morphological and physicochemical descriptors such as surface curvature, surface energy, charge, and topography [35]. In such a surface view of the protein-NM interaction, however, one has to consider the difference between the scales at which the two surfaces (i.e., protein and NM surfaces) operate (Figure 3), as is evident in the PC model. In other words, NM surface provides a relatively flat large anchoring particulate mesh (receptor) wherein protein surfaces (ligand) can potentially adhere depending on the compatibility of surface charge, shape, and possible solvation terms. This scale-difference in the two interacting partners makes such interaction analogous to, say, the docking of elongated helical peptides [36] (ligand) onto large protein surfaces (receptor), wherein complementarity in shape and surface electrostatic potential may apply as critical constraints, however, intuitively to a less degree than what has been found in both protein-protein interactions [37, 38] as well as in the folding of globular proteins to be envisaged as the docking of their interior components [39]. Generally, in a biomolecular interaction, if large molecular surfaces of proportionately equivalent size need to associate specifically, the constraints in shape and electrostatics are found to be higher as the surfaces need to be carefully tailored over large patches (say ~1600 Å^2^ for protein-protein interfaces [40] buried upon association. An equivalent comprehensive characterization of the NM-protein interface in terms of shape and electrostatic complementarities demands more structural as well as computational studies of the atomistic scale.

### 5.1. Geometric and Chemical Descriptors of Protein-NM Interaction

The affinities and amounts of proteins adsorbed on the surface of NMs are highly dependent on the composition of NM [41]. For example, the surface hydrophobicity and the availability of CH_3_ group on NM play a crucial role of PC formation around NMs. Apolipoproteins have the highest affinity for the most hydrophobic NMs, cerium oxide nanoparticle (NP), quantum dots (QD), and carbon nanotubes (CNT). Albumin, immunoglobulins, and fibrinogens bind most strongly to CNT, iron oxide particles, polymeric NP, and liposomes [13]. Transition metal (TM: Au, Cu, Ru, Rh, Pd, Ir, Pt, and Co) nanoclusters adds an indispensable major component in the repertoire of NM surfaces, wherein the n-atomic TM nanoclusters (n of the order of tens to hundreds) have been characterized to have areas of high electrostatic potential mapped along the extension of TM-TM bonds [42], traceable to the partially occupied d-orbitals.

**Figure 4:**
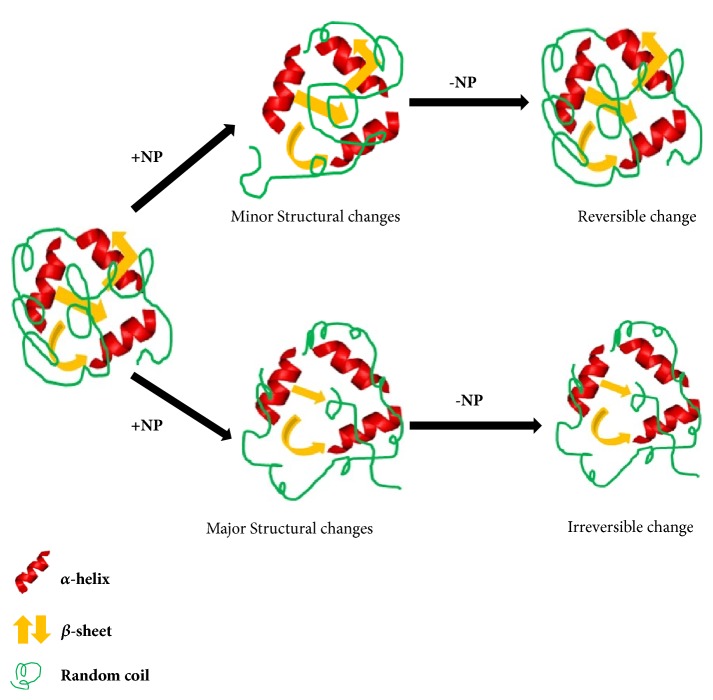
**Reversible and irreversible changes of protein by NP interaction**. Nanoparticle (NP) induced protein structural changes may result in reversible and irreversible conformational changes. The mechanism is controlled by the degree of protein structural modification. Minute structural changes of protein by NPs, which would be regained by removal of NPs, can be considered as reversible mechanism. Again significant NP-induced changes of protein structure (loss of *β*-sheets, *α*-helix), which will never be restored by removing NPs, may be termed as irreversible changes.

The NM surface curvature (flat vs. curved) is largely dependent on the cluster size and has a significant role in the absorptive properties of the corresponding surfaces which in turn controls the function of the bound proteins [13, 43]. Common blood proteins (viz., albumin, fibrinogen, *γ*-globulin, etc.) showed gradually increasing PC with increasing exposed surface of gold NPs [44]. Spherical gold NP (of say, radius of 50 nm) shows higher adsorption compared to the rod-shaped gold NP of an equivalent total surface area for having a lower surface area-to-volume ratio. The same is found with TiO_2_ nanorods and nanotubes in context of adsorbing plasma proteins [45]. These differences are also reflected in the corresponding equilibrium binding parameters (K_d_) [46]. Furthermore, surface energy of a bare NM depends on its radius of curvature since a highly curved surface will generate higher elastic stress on small area elements on the NM surface [46]. These differences in the surface architecture influence the corresponding free energy of the bound proteins as large proteins prefer to specifically pack around small surface patches, thereby resulting in larger free energies to be obtained for smaller proteins bound to an equivalent NM surface patch (Figure 4) [47]. This, in turn, may potentially influence the formation of a different corona at a higher aspect ratio of a nanomaterial (such as CNT) [48, 49]. Again, smaller nanoparticles with a higher degree of curvature will effectively have lower surface coverage, potentially interfering with protein binding. On the other hand, larger nanoparticles with lower surface curvature offer higher binding affinity for certain proteins, wherein it effectively acts as a natural chemical filter for nonspecific proteins with lesser affinity, excluding them from the PC [6, 9, 50].

## 6. Reversibility of the NM Induced Protein Conformational Changes

**Figure 5:**
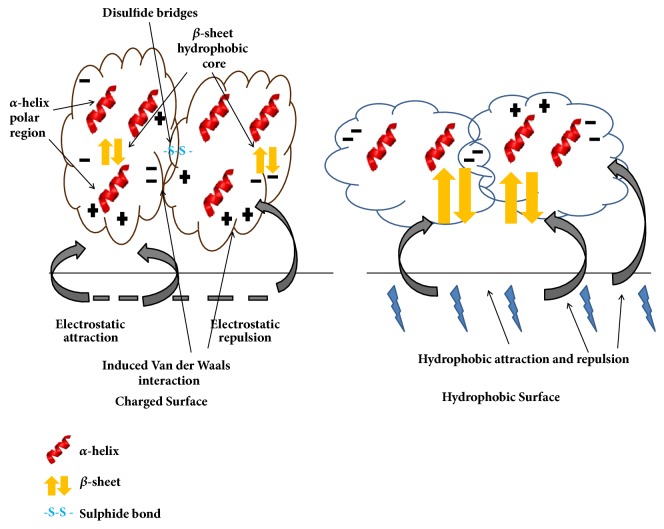
**General principles of protein adsorption on surface**. The *α*-helices and *β*-sheets are stabilized by hydrogen bonds alone and these bonds combined with hydrophobic interaction. The protein tertiary structure (the hydrophobic core) is formed by hydrophobic interaction and further stabilized by disulfide linkages. The tertiary conformation is stabilized by hydrogen bonds and electrostatic interaction between side-chain amino acid residues and reinforced by Van der Waals interaction. So, the charged surface and hydrophobic surface have major effects on protein adsorption and conformation.

One of the significant challenges pertaining to the study of NM-protein interactions is the thermodynamic characterization of the induced protein conformational changes, whether reversible or irreversible (Figure 5). This is particularly challenging given the lack of experimental structural data and appealing as protein function is directly linked to its conformation. Not all NPs bring about the same changes on the same protein and factors controlling the surrounding chemical environment like pH, further influencing the changes. The conformational changes also vary as a function of the binding site. For example, BSA upon adsorption to gold NPs displays more stable and rigid conformational changes (irreversible) at both secondary and tertiary structural level while more flexible (reversible) changes are encountered for the same protein adsorbed to the boundary surface of gold NPs [20]. Again, virtually no conformational change was detected on BSA upon adsorption to C60 fullerene while only marginal changes were obtained upon binding to ZnO-NPs [51]. Furthermore, a dose dependence on the induced changes was also found in the case of BSA [52]. On the other hand, irreversible changes were detected in albumin and lacto peroxidase upon adsorption to silica NP while RNAse and lysozyme retained their native structure when adsorbed to the same NP [53]. Again, in systems like CuO-BSA, Fe_2_O_3_-Hb, and SWNT-Lyz no remarkable changes were detected [23, 24, 54].

### 6.1. Protein Conformational Changes as a Function of Amino Acid Composition

The conformational changes are reflected in the differential attachment of amino acids to the NPs. For example, amino acids with small side-chains (e.g., Gly, Ala, Val, Ser, and Cys) have been found to attach to larger NPs with a greater propensity. On the other hand, the flexibility has been found to be greater for proteins containing amino acids with elongated side-chains (e.g., Lys, Arg, and Met). Some amino acids (such as Ala, Cys, Asp, Pro) have been found at the protein-NM interface with greater propensities, while some of them (Cys, Leu, Ala, and Ser) have been probed to have a more determining role in the recognition of the appropriate NM surface. Some of these amino acids again have preference towards helices (e.g., Leucine) which make sense as helices are more prone to attach to NPs due to their inherent hydrophilicity [55]. Myoglobin and BSA (both helix-rich proteins) showed conformational changes upon adsorption onto Si-NP in a dose dependent manner [56], wherein geometry dependent double layer may play a crucial role for myoglobin denaturation. On the other hand, ß-casin, ribonuclease-A, and lysozyme did not show any conformational changes upon binding to NPs of any size [56]. Also, higher protein coverage on NM surface could invoke molecular crowding which may potentially affect protein folding as well as the activity of the bound protein(s). For example, in the case of adsorbed cytochrome-C on CoFe_2_O_4_ NP, higher coverage did not change the protein structure but changes were found for lysozymes bound on Si-NP surfaces [57] (Table 2).

### 6.2. A Structural Inner-View of the Induced Conformational Changes

**Figure 6:**
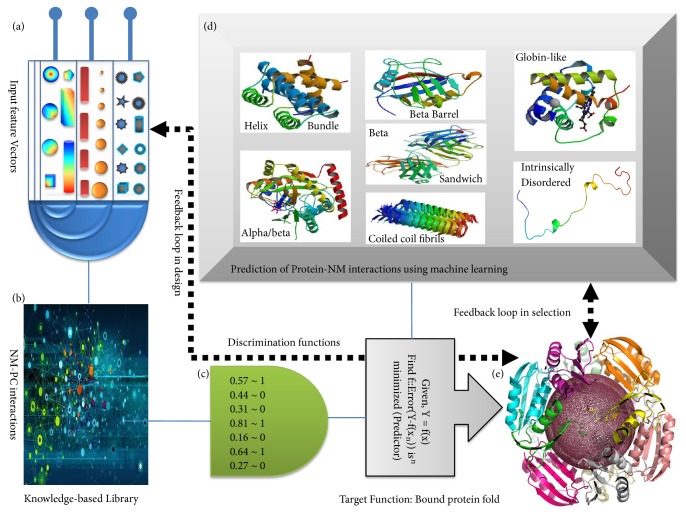
**Artificial intelligence in NM-protein interactions**. The future translational applications of nontechnology in human body will greatly be influenced by NM-protein interactions. Exploring the instinctive and automatic prediction of nanosurface interactions with protein using machine learning will help in estimating the risk profiles of using NM and also the feedback can guide us designing the desired NM that facilitates beneficial interactions. The unique NM features ((a) size, shape, granularity, pH, etc.) that are hypothesized to contribute to NM-protein interactions could be considered as (a nonrigid set of) “input feature vectors” restoring the knowledge from available experimental data (b). The set of features can then be trained against appropriate target functions to build machines (c). Different combination of features needs to be attempted till optimization. This could give us a hypothesis as to whether or not (or in what extent) the NM will interact with specific/nonspecific proteins (d). A successful endeavour of this exercise should further be taken into the prediction and design of beneficial nanoprotein interactions. It should be noted with care that the training and cross-validation of such machine learning based predictors is a long-term exercise falling under continuous evaluation (on new and updated datasets as they get available) demanding an ever-increasing accuracy until it is “adequate” (e).

When the hydrophobic core of a globular protein, enclosed by *α*-helices and ß-sheets, approaches an NM, the NM surface replaces water molecules attached to the protein surface (Figure 6) [7]. As a consequence, the percentage of *α*-helices is decreased while the percentage of *β*-sheets is increased for helix-rich proteins like HSA having arguably hydrophilic surfaces. The rearrangement of the hydrogen bonds plays the key role in such cases, wherein more ordered intrachain hydrogen bonds are dissolved within *α*-helices compensated by reformation of less ordered hydrogen bonds between *α*-helices and NMs and thereby leading to a net gain in entropy [7]. Again, for arguably hydrophobic NM surfaces, the hidden nonpolar parts of the protein often get exposed leading to another type of conformational change [41]. For example, there has been no disruption of the overall protein structure while there is adsorption of ß-sheets on gold NP [58] and while a well-structured collagen molecule undergoes high conformational changes upon interaction with hydrophilic Si-NP surfaces. A reversible structural rearrangement was found to occur on lysozymes adsorbed on polar Si-NP surfaces, wherein the native structure was possible to be restored after removal of the NPs [59]. As per an associated surface denaturation concerned, the working hypothesis is that the mechanisms are different at low coverage (where high level of available surface area promotes unfolding) than at high coverage (where crowing effects and steric hindrances prevent unfolding).

## 7. Protein Induced NP Aggregation

The binding of proteins onto NMs can result in NM aggregation along with protein aggregation. It has been shown that protein self-assembly in the presence or absence of NMs is highly controlled not only by proteins' properties, like aggregation rate and inner stability, but also by the physiochemical properties of NMs, like shape, size, and NMs/protein ratio. Upon interaction with NMs, some proteins predominantly consist of ß-sheet structure and tend to self-assemble inside, whereas others get aggregated randomly [8]. For example, lysozyme-gold NPs formed assembly when they were mixed and underwent protein conformational change on gold NPs. The surface bound lysozyme interacted with other proteins in the solution followed by gold NP aggregation. Positively charged part of protein gets exposed in solution when it adsorbed onto a NM surface. This phenomenon depends on the 3D structure and the position of lysine and arginine residues [60]. Lowering the pH below the pI value of a protein can encourage NMs aggregation. 6-mercaptopurin, citrate, *ω*-mercaptoundecanoic acid, surface stabilizing molecules, etc. are known to influence the NMs aggregation as they have different pKa values from the NMs surface. Their dissociation from the surface affects the aggregation of NMs after partial adsorption of protein onto NMs surface. Protein mixed with NPs usually plays crucial role in the cluster formation [8, 61]. Amyloidogenic peptides influence protein-NP aggregation by interacting with other peptides and formation of nuclei for protein-NP aggregation triggered through the combination of long range electrostatic repulsion and short-range attraction between proteins bound to NPs [8, 61].

## 8. Impact to the Biology of the Targeted System

The NP-induced reversible conformational changes also affect the downstream protein-protein interaction cascade, which, in turn, influences cellular signaling and eventually transcription. Also, such changes can increase the accessibility of the active sites of certain enzyme(s) towards NPs [8]. To be more specific, NP-protein binding has two major consequences. Generally, irreversible changes to the protein lead to a reducing bioactivity of the concerned protein while changes that level up to scale the protein energy in a controlled way favour the overall functionality of the system. For instance, Si-NP was able to induce a molten globule like structural change in carbonic anhydrase, while upon removal of the NPs three intermediate native-like conformations were obtained and the catalytic activity of the enzyme was retained [60]. It is known that NP-protein corona (PC) rather than bare NP determines the NP-Cell interactions, including endocytic pathway and biological responses. The experiments for endocytosis specify the fact that different endocytic pathways might be responsible for the alternative roles of PC in the interaction of sized NPs with different cell lines. The therapeutic and toxicological profiles of a NP might be altered by altering cell uptake as well as cellular response through NP-PC incorporation. It was observed that, in the presence or absence of a PC, silica NP showed different uptake efficiencies. AuNP particle size and surface ligand grafting density can also affect the adsorption of serum proteins and cell uptake in macrophages [62]. Covalently bound horse radish peroxidase, substilisin, Carlsberg, etc. were also shown to retain their catalytic activity and native structure after removal of SWNT from the bound form [60]. One of rare but important factors on the composition of PC is the changes in incubation temperature of the NPs. For instance, plasmonic heat induction changes composition of hard corona adsorbed on gold nanorods which was further supported by computational molecular modeling studies [61].

### 8.1. Possible Deleterious Effects

Conformational changes in certain proteins may also trigger insoluble aggregation and eventually fibrillation. Generally, the enormous surface area presented by naked NPs decrease the lag time for the formation of a protein nucleus to enhance the rate of protein fibrillation [9]. On the other hand, the same NPs (e.g., silica, polystyrene, and CNT) when surrounded by PCs inhibit fibrillation [12, 63]. Fibrillation (in ß2-microglobulin) was also found to be induced by other NMs like copolymer, ceria, CNT, and QD due to the increased protein localization on NM surface resulting in the formation of oligomers [63].

Electron confinement and the formation of electron hole pairs (Supplementary) at the NM surface may also lead to the undesired breakage of covalent bonds or cross-links in protein SH domains [6]. For instance, chicken egg lysozyme bound to SiO_2_-NP surfaces was found to induce unfolding of a critical *α*-helix resulted in the loss of its catalytic property [6].

### 8.2. Possible Benefits

Interaction with NP can sometime serve as protective layers against thermal disruption of protein secondary structure. Mukhopadhyay et al. showed that bacterial mesophilic laccase [64] treated with Cu_2_O-NPs retains its secondary structure even at temperature of ~ 80-90°C while the untreated one became disrupted (reflected in a substantial decrease of its *β*-sheet content). Almost an identical result was found in case of a protease, wherein, hydroxyl-apatite NP helped to retain its secondary structure at higher temperatures [64–68] (Table 3).

## 9. Outstanding Questions

Till now the structural modification of proteins upon NMs interaction have been shown by most of the literature in* in-vitro* studies. Still there is not much evidence of protein conformational changes due to NMs interaction after nanobiosystem is taken up by living organisms. So, the obvious question is what would be the fate of protein as well as NMs in an* in-vivo* study?

Most of the studies were based on the adsorption of protein onto NMs to discuss the effect. But apart from adsorption, there are other modes of NM-protein interaction, like cross linking, entrapment, encapsulation, self-assembly, etc. So, the effect on protein structures for these alternative* modus operandii* of nanoprotein interactions is another essential query that needs to be addressed adequately.

There are very few instances where NM induced enzymatic activity due to interaction. If nanomaterials can stabilize a protein structure, they may also potentially hyperactivate the enzyme by changing its secondary structure/modulating its folding to expose more catalytic sites. So, what are the other possibilities of functional gain of a protein due to NM interaction? Apart from aggregation, what other structural and functional modulations may be experienced in NMs due to protein interaction? And lastly, is it possible to use engineered protein or surface stabilizing agents for NMs to minimise the structural damage and aggregation?

## 10. Conclusion and Perspective

Characterization and analysis of proteins bound to NM surface are an important step to understand the effect of NM-PC for biological and medical applications. Research has thus characterized the physical chemistry of PC formation and associated protein conformation. One of the insightful ideas was the ‘memory of PC formation.' Again, the potential consequences of NM-protein interaction are the alteration of biomolecular structure. Conformational change may affect protein function, e.g., enzymatic activity. However, the changes can also be utilized to good effects as exemplified in the case of serving as a thermostable protective layer for certain mesophilic enzymes. Aggregation of NP can potentially change its interaction pattern affecting the normal cell homeostasis. Interestingly, most studies about NM-protein interactions are done* in-vitro*. So, understanding the behavior of NM-protein complexes* in-vivo* remains yet to be explored, which is a challenging task. Another current lacuna in the field is the lack of enough high-resolution structural techniques which throws an open challenge to the computational structural biology community to model the interaction and dynamics such large and complex macromolecular assemblies. But in some current simulation methods, advances in the understanding of chemical bonding, in the development of force fields, and in the development of chemically realistic models are described [69]. For example, an introduction of the silica models or hydroxyapatite model provides full validation for interfaces [70, 71]. A successful endeavour towards this direction will also enable the study of the surface architecture of NM-PC complexes in atomistic details. This is of utmost importance to decipher the scope of the most popular concept of ‘complementarity in biomolecular recognition' in such complexes. Also, from a computational end, it cannot be left overlooked that there is a significant scope of the ever-so-successful machine learning approach [72, 73] to be implemented to predict the yet unexplored NM-protein interactions (Figure 6) once enough high-resolution structural and binding data are available by either experimental or computational means.

The implementation of the “artificial intelligence” approach to the nanoprotein system is currently only hypothetical, however, once grown enough matured, it appears perfectly rational to imagine instinctive prediction of nanosurfaces, potentially facilitating beneficial interactions, with a concomitant estimation of the corresponding risk profiles. The machine learning approach has been extremely successful across various branches of informatics and there is no reason to believe that it won't be successful here as well. Overall, emergence of the computational structural field in probing NM-protein complexes should definitely foster future translational applications of nontechnology in the human body as the subject is heavily dependent on NM-protein interactions.

## Figures and Tables

**Table 1:** Main forces governing the interfacial interaction between NM and biomolecule [5].

**Force **	**origin and nature**	**range (nm)**	**Possible impact on the interface**
**Hydrodynamic interactions**	Convective drag, shear, lift and Brownian diffusion are often hindered or enhanced at nanoscale separations between interacting interfaces	10^2^ to 10^6^	Increase the frequency of collisions between nanoparticles and other surfaces responsible for transport

**Electrodynamic interactions**	VDW interactions arising from each of the interacting materials and the intervening media	1 to 100	Universally attractive in aqueous media; substantially smaller for biological media and cells owing to high water content

**Electrostatic interactions**	Charged interfaces attract counter–ions and repel co–ions through Coulombic forces, giving rise to the formation of an electrostatic double layer	1 to 100	Overlapping double layers are generally repulsive as most materials acquire negative charge in aqueous media, but can be attractive for oppositely charged materials

**Solvent interactions**	Lyophilic materials interact favourably with solvent molecules	1 to 10	Lyophilic materials are thermodynamically stable in the solvent and do not aggregate
	Lyophobic materials interact unfavourably with solvent molecules		Lyophobic materials are spontaneously expelled from the bulk of the solvent and forced to aggregate or accumulate at an interface

**Steric interactions**	Polymeric species adsorbed to inorganic particles or biopolymers expressed at the surfaces of cells give rise to spring–like repulsive interactions with other interfaces	1 to 100	Generally, increase stability of individual particles but can interfere in cellular uptake, especially when surface polymers are highly water-soluble

**Polymer bridging interactions**	Polymeric species adsorbed to inorganic particles or biopolymers expressed at the surfaces of cells containing charged functional groups can be attracted by oppositely charged moieties on a substrate surface	1 to 100	Generally, promote aggregation or deposition, particularly when charge functionality is carboxylic acid and dispersed in aqueous media containing calcium ions

**Table 2:** Summary of literature on proteins subjected to conformational changes upon interaction with nanoparticle surfaces [7, 8].

**NP type and size**	**Protein investigated**	**Change in protein structure**	**Analytical technique**	**Observations**
**ZnO NPs (25 nm)**	*Vibrio cholera* Tox r	Yes	CD	NP-protein complex susceptible to denaturation

**ZnO NPs (N/A)**	BSA	Yes	CD	Minor conformational changes, secondary structure retained

**ZnO NPs (N/A)**	BSA	Yes	FTIR	Minor conformational changes in secondary structure

**TiO2 NPs (20 nm)**	Tubulin	Yes	FS	Protein polymerization affected

**SiO2 NPs (**~**40) nm**	BSA	Yes	RS	BSA and lactoperoxidase bound irreversibly
	Hen egg lysozyme	No		
	RNASe A	No		
	Lactoperoxidase	Yes		

**SiO2 NPs (6,9,15 nm)**	Human Carbonic anhydrase	Yes	NMR	Protein activity was retained

**Alumina and hydroxyapatite Particles(100-300nm) **	BSA	Yes	FTIR	Loss in *α*-helical structure
	Hen egg lysozyme	Yes		
	Bovine serum fibrinogen	Yes		

**Gold (45 nm)**	BSA	Yes	CD	Conformational change was dose dependent

**Gold (5-100 nm)**	Albumin	Yes	CD and FS	Minor conformational changes observed
	Fibrinogen	Yes		
	*ɣ*-globulin	Yes		
	Histone H3	Yes		
	Insulin	Yes		

**Gold (7-22 nm)**	Human Fibrinogen	Yes	CD	Unfolding induced immune response in THP-1 cells

**SPIONs (5-10 nm)**	Transferrin	Yes	CD	Irreversible interaction

**SWCNTs (N/A) **	Horse radish peroxidise	No	CD	NP-protein complexes retained enzymatic activity
	Subtilisin Carlsberg	No		
	Chicken egg white lysozyme	No		
	Laccase	Yes		

**Table 3:** Secondary structure change through NM-protein interaction.

**Secondary structure change**	**NM-protein complex**
α**-helix increases**	C60-HSA, SWNT-laccase.

]α**-helix decreases**	Graphene-QDs-HSA, CdTe-QDs-HSA, Mercapto propionic acid-CdTe-QDs-HSA, Glutathione- CdTe-QDs-HSA, CDSe/ZnS-QDs-HSA, L-Cys capping CdTe-QDs-Lyz, CdS NP-HSA, CdS NP-BSA, N-Acetyl-L-cysteine-capped CdTe–BSA, N-Acetyl-L-cysteine-capped CdTe–BHb, N-Acetyl-L-cysteine-capped CdTe–catalase, CdTe–*α*-chymotrypsin, Cu–BSA, CuO–ß-galactosidase, ZnO–HSA, ZnO–lysozyme, ZnO–*α*-lactalbumin, ZnO–ToxRp, Ag–PVT–HSA, Ag–BHb, Ag–urease, Histidine capped Au-NPs–BSA, Au-NPs–BSA, *γ*-Fe2O3–fbrinogen, NH_2_–Fe_3_O_4_–BSA, Fe_3_O_4_–BSA, Fe_3_O_4_–tubulin, OH–MWCNTs–transferring, OH–MWCNT–BSA, Carboxylated-MWCNT–BSA, Carboxylated-SWCNT–BSA, OH–MWCNTs–Hb, OH–SWCNTs–Hb, OH–MWCNTs–Mb, OH–SWCNTs–Mb, NH_2_–PAMAM–insulin, CHP–HSA, CHCP–HSA

**ß-sheet increases**	SWCNT–tau protein, ND–Hb, SWNT-laccsae, HAp-protease.

**No change**	C60-BSA, L-Cys capping CdTe-QDs-BSA, CuO–BSA, Fe_2_O_3_–Hb, SWNT–Lyz, Oxidized SWNT–Lyz, MWCNT–tau protein, COOH–PAMAM–insulin, OH–PAMAM–insulin.
